# Knowledge, attitude, and practice of medication therapy management: a national survey among pharmacists in Indonesia

**DOI:** 10.3389/fpubh.2023.1213520

**Published:** 2023-07-17

**Authors:** Farida Rendrayani, Sofa Dewi Alfian, Wawan Wahyudin, Irma Melyani Puspitasari

**Affiliations:** ^1^Department of Pharmacology and Clinical Pharmacy, Faculty of Pharmacy, Universitas Padjadjaran, Sumedang, Indonesia; ^2^Center of Excellence for Pharmaceutical Care Innovation, Universitas Padjadjaran, Sumedang, Indonesia; ^3^Ciloto Health Training Centre, Ministry of Health Republic of Indonesia, Cianjur, Indonesia

**Keywords:** medication therapy management, knowledge, attitude, practice, pharmacists, community health centers, Indonesia

## Abstract

**Introduction:**

The use of medication therapy management (MTM) is a proven method for reducing medication errors. MTM services rely heavily on pharmacists as service providers, particularly in community health centers (CHCs). Thus, understanding the knowledge, attitudes, and practices (KAP) of MTM among pharmacists in CHCs is crucial to the strategy for the implementation of MTM program in Indonesia. This study aimed to assess the level of KAP regarding MTM among pharmacists working at CHCs and its associated factors and investigate pharmacists’ perceptions of the barriers and facilitators of MTM provision in the future.

**Methods:**

A cross-sectional online survey was conducted. The respondents were pharmacists working at CHCs in 28 provinces in Indonesia. Descriptive statistics were used to summarize the responses. Demographic differences were determined using Chi-square and Kruskal–Wallis tests, and associations were identified using multivariable ordinal regression for knowledge and multivariable logistic regression for attitude and practice. Barriers and facilitators were determined from codes and categories of frequency derived from pharmacists’ responses to the open-ended questions.

**Results:**

Of the 1,132 pharmacists, 74.9% had a high level of knowledge, 53.6% had a positive attitude, and 57.9% had a positive practice toward MTM. Gender, practice settings, province of CHCs, years of practice, and experience in MTM services were factors associated with the KAP level. Respondents perceived that the chronic disease conditions in Indonesia, MTM service features, and current practices were facilitators of MTM provision. The lack of interprofessional collaboration, staff, pharmacist knowledge, patient cooperation, facilities/drug supply/documentation systems, stakeholder support, and patient compliance were the most common barriers to MTM implementation in the future.

**Conclusion:**

Most of the pharmacists had high knowledge of MTM; however, only half had positive attitudes and practices toward MTM. Information about factors associated with the KAP level suggests that direct involvement is essential to improve pharmacists’ understanding and view of MTM. Pharmacists also perceived barriers to the MTM provision in the future, such as interprofessional and pharmacist-patient relationships. A training program is needed to improve the KAP of MTM and develop skills for collaborating with other healthcare professionals and communicating with patients.

## 1. Introduction

As the leading cause of morbidity and mortality worldwide, medication errors are becoming a global issue (1, 2). It is defined as any error during the medication use process whether in the planning or in the execution of that plan (2). Medication error threats patients’ safety, and the risk of medication error becomes higher in chronic diseases patients due to comorbidities and polypharmacy (2, 3).

The risk of medication error can be reduced effectively through a mechanism of medication therapy management (MTM) (1, 4). Its implementation follows a model framework that comprises five key elements: medication therapy review, medication-related action plan, interventions and referrals, and documentation and follow-up (5). In 2017, Indonesia adopted the MTM program to improve the quality of chronic disease care (6). In collaboration with the Social Insurance Administration Organization (BPJS), the government has piloted the program at several community health centers (CHCs), which serve as gatekeepers for patients before referral to hospitals for further treatment (6–9). Studies have shown the positive effect of this pilot project of MTM on clinical and humanistic outcomes (6–8). These findings are consistent with those of many studies that have demonstrated the benefits of MTM on clinical, economic, and humanistic outcomes in other countries (5, 10).

The implementation of MTM requires the qualification of all health professionals, including pharmacists (1). Pharmacists’ competency, which includes knowledge, attitude, and practice (KAP), contributes to MTM success (11, 12). Therefore, the assessment of KAP regarding MTM is essential to the strategic plan for effective program implementation (10). To the best of our knowledge, studies on the KAP toward MTM and its factors in Indonesia are limited. Only one study assessed Indonesian pharmacists’ perceptions regarding intention and readiness to provide MTM services (13). The study also did not report the coverage of its respondents. This study aimed to assess the level of KAP regarding MTM among pharmacists working at CHCs and its associated factors and investigate pharmacists’ perceptions of the barriers and facilitators of MTM provision in the future. The study was a national survey designed to obtain a better overview of the issue.

## 2. Materials and methods

This study followed the Checklist for Reporting of Survey Studies (CROSS) guideline for its reporting (14). The CROSS checklists of this study are presented in Supplementary material (Supplementary Table S1).

### 2.1. Study design

This cross-sectional study was conducted as an online survey of pharmacists in 28 provinces of Indonesia. Pharmacists who worked at CHCs and consented to participate were included in the study. Those who did not finish the questionnaire were excluded. The networking of the Indonesian Pharmacists Association (Ikatan Apoteker Indonesia/IAI) and Public Health Pharmacy (Himpunan Seminat Farmasi Kesehatan Masyarakat/Hisfarkesmas) were utilized, formally or personally, to recruit a convenience sample of pharmacists. Participating pharmacists received two participation credit units from the IAI as a token of appreciation.

### 2.2. Sample size

Using the Slovin formula, a minimum of 436 participants was required to obtain a 95% confidence level (5% margin of error) with an unusable response of 10% for the population size of 18,958 based on the total number of pharmacy staff at CHCs in Indonesia (15–17). The minimum sample was also calculated for each of the 28 provinces based on the proportion of each region to the total population.

### 2.3. Study instrument

We modified the instrument developed by Al-Tameemi and Sarriff based on literature and conducted a thorough discussion to meet the study objectives and context (4, 18, 19). Backward and forward translations were conducted at the West Java Provincial Language Centre (Balai Bahasa Jawa Barat) to obtain a version in Bahasa. A panel of three experts (a health professional trainer, a senior pharmacist at CHC, and a researcher of pharmacotherapy and patient care) validated the content of the translated questionnaire. The second round of content validation yielded a Lawshe content validity ratio of 1 for all items, indicating that the content was valid (20, 21). Then, the questionnaire was piloted in two rounds, each on 30 pharmacists from several provinces of Indonesia (1st round: 76.67% from West Java and 6.67% from Central Java; 2nd round: 26.67% from Central Java, 13.33% from South Sumatera, and 10% from West Java) (22). The 2nd round showed that the questionnaire was valid (all items have Pearson r > 0.361 in the construct validity test) and reliable (Cronbach’s α of KAP sections >0.600) (23–25). Therefore, the final questionnaire consisted of six sections: eight questions on sociodemographic data, six on knowledge, 10 on attitudes, eight on practice, two open-ended questions about barriers and facilitators, and nine additional information regarding practice.

Demographic data were collected from every participant, including gender, age, educational background, practice setting, and years of practice. Regarding the practice setting, we asked whether the Community Health Center was outpatient/inpatient, located in what province, and provided the drug of the government program.

In assessing knowledge about MTM, respondents were asked one question about their understanding of the pharmacological and non-pharmacological therapies covered by MTM and five questions about the specific activities of the MTM core elements. Each correct answer scored 1 point, whereas an incorrect answer scored 0. Then, the sum of the knowledge score was calculated for each participant, which ranged from 0 to 6.

Respondents’ attitudes were measured based on the agreement with 10 positive statements regarding pharmacists as primary providers of MTM services, pharmacists’ role in each core element of MTM, benefits of MTM services, expansion of the role of pharmacists, and competencies required to provide the service. A 5-point Likert scale was used for the attitude section (strongly disagree = 1, strongly agree = 5). Thus, the maximum possible score was 50.

Pharmacists’ practice toward MTM was evaluated by examining daily activities that support MTM provision, including the use of medical records as a means of communication between health workers, specific review of medication therapy, designing strategies to deal with medication-related problems, collaboration among health professionals, and documentation of services/interventions provided. We used yes/no/not sure questions, which scored 1 for the yes answer and 0 for others. Therefore, the maximum possible score was 8 for the practice section.

Two open-ended questions were added, asking pharmacists about what could facilitate or hinder the implementation of MTM in the future. Eight additional questions were also added regarding the intention to provide MTM service: availability of time to provide MTM, availability of time and physical space for counseling, accessibility of guidelines, and training needs. Moreover, yes/no/not sure questions were used, which scored 1 for the yes answer and 0 for others. The sum scores for this section were not calculated. The matrix of the final questionnaire is available in Supplementary material (Supplementary Table S2).

### 2.4. Data collection

The survey link was distributed using a digital leaflet, and data were collected using Qualtrics® (Provo, USA). The survey was accessible from October 11 to November 11, 2022. In the initial section, the respondents had to provide their IAI ID numbers. This ID could prevent participation by individuals who were not pharmacists and was beneficial in examining duplicate submissions. However, they were informed that their responses would be processed and presented anonymously. Participants should click the [Agree] button before taking the survey to indicate their consent to participate in this study.

### 2.5. Ethical approval

The study received ethical approval from the Research Ethics Committee of Universitas Padjadjaran (No. 604/UN6.KEP/EC/2022). The first page of the survey provided information about the research and allowed pharmacists to decide whether to participate. Clicking [Yes, I agree.] would prompt them to complete the questionnaire, whereas [No, I do not agree.] would lead them to the end page. In addition, pharmacists could opt out of finishing the survey by not clicking on [Submit my response].

### 2.6. Data analysis

First, the data distribution was analyzed before performing other statistical analyses. If the data did not follow a normal distribution according to the Kolmogorov–Smirnov test, we used the median for KAP categorization, which split KAP into high/positive if the scores were greater than or equal to the median and vice versa (26–28). It is necessary to categorize the results for ease of interpretation and follow-up (29), especially in clinical practices. A sensitivity analysis was conducted to determine whether changes (±10%) in the categorization cutoff would affect the study findings and conclusions (30).

Before analysis, weighting by complex sample analysis techniques in SPSS was performed for the province variable (31). Weighting was needed to statistically correct the unequal proportion observed during sampling (32). Descriptive statistics were utilized to describe demographics, KAP, and barriers and facilitators of the MTM provision. A Chi-square or Kruskal–Wallis test was employed to analyze the association between each sociodemographic factor and KAP level. Multivariable ordinal regressions were performed to identify factors associated with knowledge of MTM simultaneously, whereas multivariable logistic regressions were conducted for the attitude and practice factors. IBM SPSS Statistics version 27.0 (IBM Corp., New York, USA) was used for the statistical analysis.

Codes emerging from pharmacists’ responses to the open-ended questions about barriers and facilitators toward MTM provision in the future were analyzed. Grouping codes were categorized, and the frequency was calculated. NVivo version 11 (QRS International Pty Ltd., Victoria, Australia) was used for the qualitative data analysis.

## 3. Results

### 3.1. Respondents’ characteristics

**Figure 1:**
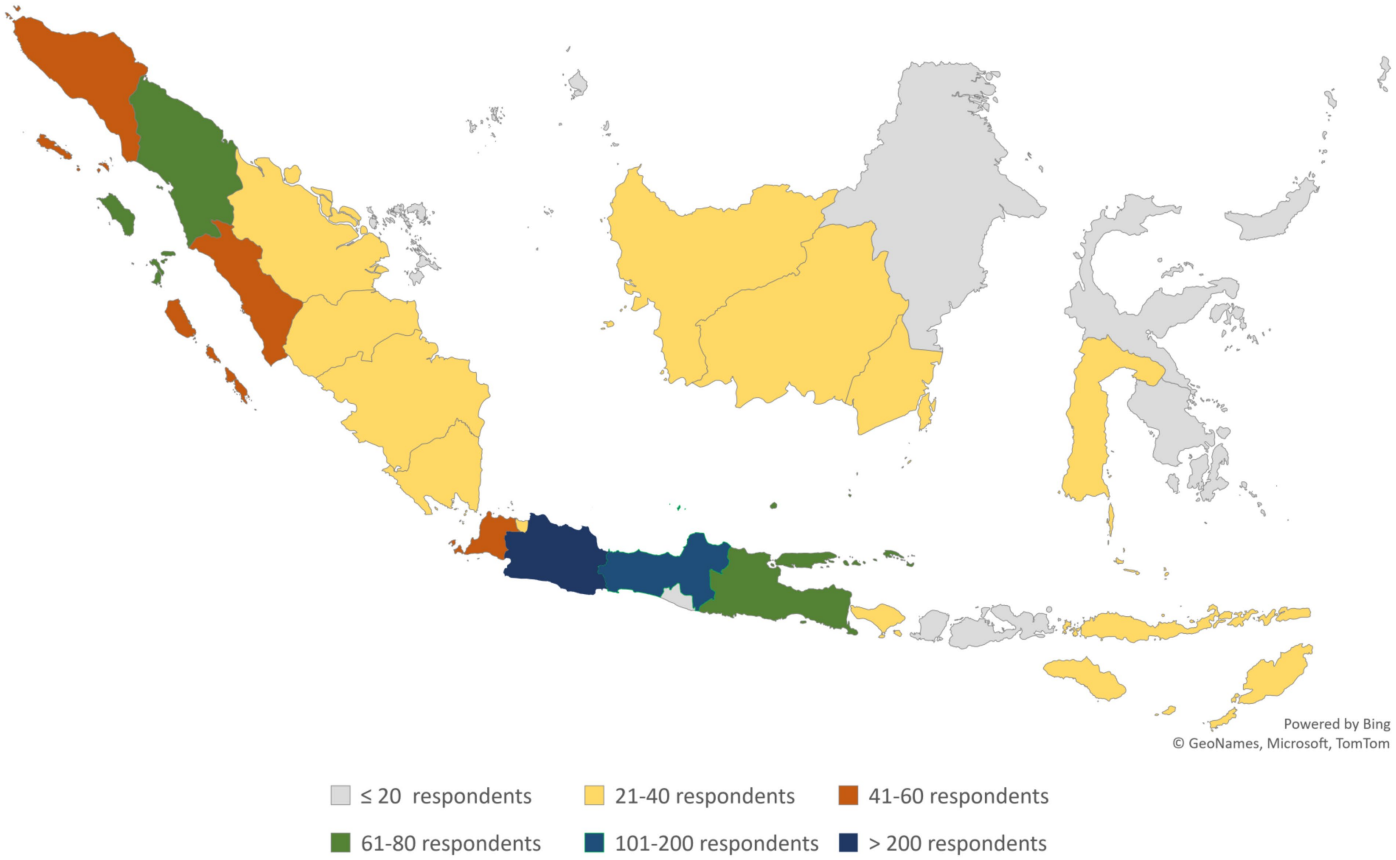
Pharmacists’ distribution by province of CHCs.

The survey successfully included responses from 1,132 pharmacists in 28 provinces of Indonesia. The majority of respondents were women (78.4%), aged 20–30 years (51.6%), with pharmacist professional backgrounds (95.9%), working in outpatient CHCs (59%), working in CHCs that provide the drug of the government program (97%), with a length of practice of 0–10 years (90.1%), and have ever provided MTM service (50.2%). Table 1 presents the respondents’ demographic characteristics, and Figure 1 shows the pharmacists’ distribution according to the province of CHCs.

**Table 1:** Sociodemographic characteristics of the respondents (*n* = 1,132).

Characteristics	Unweighted		Weighted
	*n*	%	% (95% CI^1^)
Gender			
Men	245	21.6	21.6 (19.0–24.4)
Women	887	78.4	78.4 (75.6–81)
Age (years)			
20–30	578	51.1	51.6 (48.3–54.8)
31–40	468	41.3	41.5 (38.3–44.8)
41–50	74	6.5	6.1 (4.8–7.9)
>50	12	1.1	0.8 (0.4–1.5)
Educational background			
Pharmacist professional	1,089	96.2	95.9 (94.3–97.1)
Master	43	3.8	4.1 (2.9–5.7)
Doctoral	0		
Practice settings			
Inpatient	421	37.2	41.0 (37.9–44.1)
Outpatient	711	62.8	59.0 (55.9–62.1)
The Community Health Center provides the drug of the government program			
No	1,095	96.7	97.0 (95.7–97.9)
Yes	37	3.3	3.0 (2.1–4.3)
Years of practice			
0–10	1,017	89.9	90.1 (88–91.9)
11–20	99	8.7	8.7 (7.1–10.8)
21–30	11	1	0.6 (0.3–1.3)
>30	5	0.4	0.5 (0.2–1.3)
Have ever provided MTM services			
No	561	50.4	50.2 (46.9–53.5)
Yes	571	49.6	49.8 (46.5–53.1)

1 CI, confidence interval.

### 3.2. Pharmacists’ knowledge of MTM

Table 2 presents the proportion of correct answers to knowledge regarding MTM. Most respondents correctly answered the question regarding the documentation of MTM services (91.60%). Approximately 85% of pharmacists correctly answered the questions about personal medication records and MTM beneficiaries. In addition, pharmacists appeared to need more understanding of the comprehensive and targeted medication therapy review and the medication-related action plan.

**Table 2:** Pharmacists’ knowledge of MTM.

No.	Items	Correct Answers		
		Unweighted		Weighted
		*n*	%	% (95% CI^1^)
1.	Any patient who uses prescription and non-prescription medications, herbal products, or other dietary supplements could potentially benefit from MTM.	975	86.1	85.9 (83.4–88.0)
2.	Patients may receive a comprehensive medication therapy review once a year and a targeted medication therapy review if there are new medication-related issues.	851	75.2	75.7 (72.8–78.4)
3.	In MTM service, the personal medication record serves as a tool for patients to manage their treatment based on the pharmacist’s instructions.	986	87.1	85.8 (83.2–88.1)
4.	Patients are actively involved in addressing medication-related problems by referring to the medication-related action plan records that pharmacists make.	908	80.2	79.4 (76.5–81.9)
5.	Pharmacists’ intervention to the medication therapy could be performed during MTM visits.	957	84.5	84.9 (82.3–87.1)
6.	The documentation of MTM services includes recording the schedule of control visits, amount of time with patients, and feedback on health workers or patients.	1,042	92.0	91.6 (89.5–93.3)

1 CI, confidence interval.

**Figure 2:**
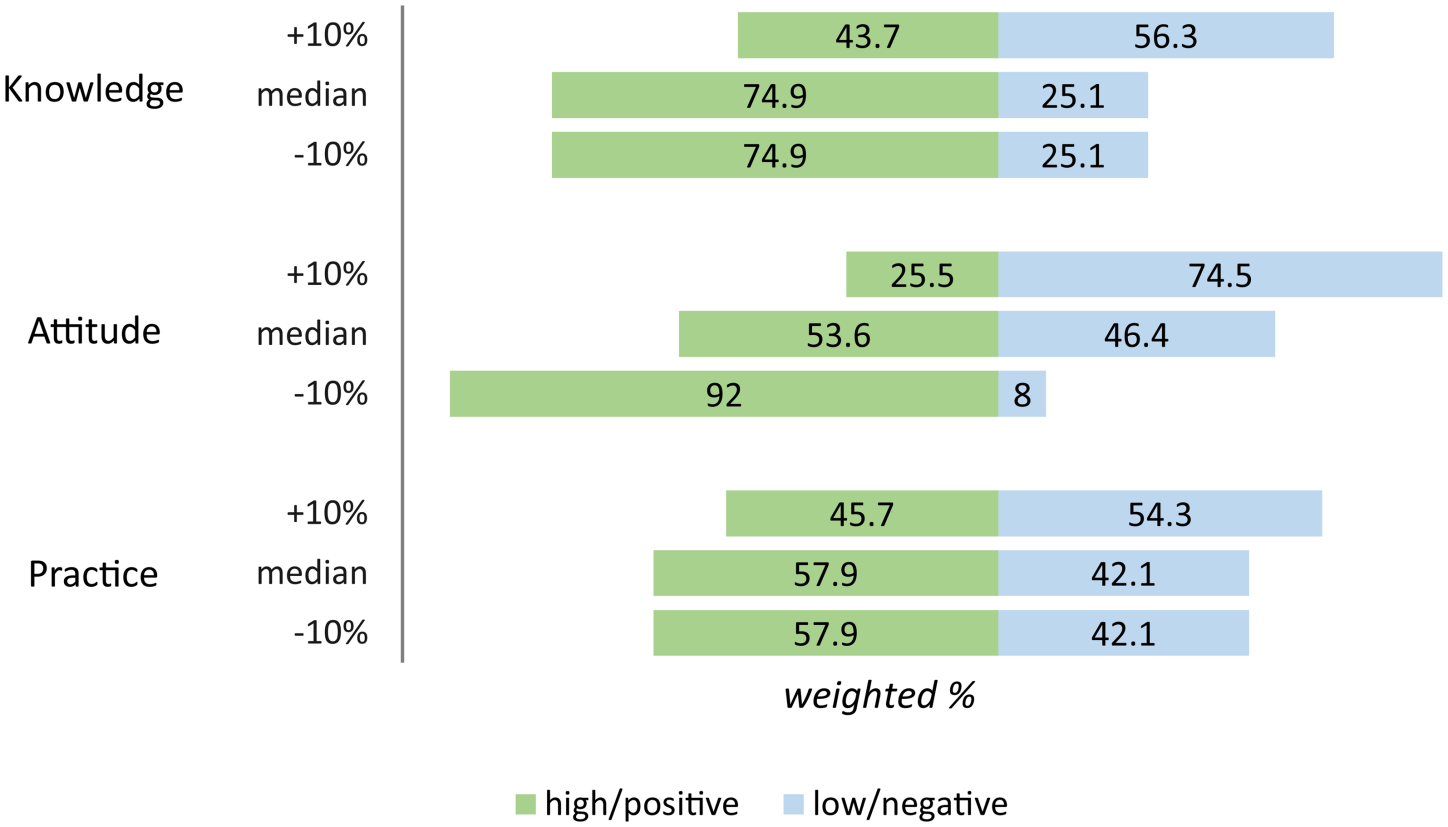
Sensitivity analysis of the cutoff value.

The median of the total knowledge score was 5 (min. = 0, max. = 6) out of 6. After the median split, 74.9% of the respondents were categorized as having high knowledge of MTM. Sensitivity analysis showed that lowering 10% of the cutoff value did not change the finding. However, an increase of 10% resulted in a lower frequency of respondents with high knowledge than those with low knowledge (Figure 2).

### 3.3. Pharmacists’ attitude toward MTM

Table 3 illustrates the responses of the respondents in the attitude section. More than 85% of the respondents indicated approval of all statements provided. Most respondents agreed that reviewing patient medication and providing interventions were the essential roles of pharmacists in patient drug therapy (96.60%). Respondents also agreed that creating a personal medication record could help avoid medication-related problems (96.20%). Respondents agreed that the MTM provision is a unique opportunity for pharmacists to expand their role (94.30%) and that their application requires more knowledge (93.15%).

**Table 3:** Pharmacists’ attitude toward MTM.

No.	Items	Answers				
		Unweighted %, Weighted % (95% CI^1^)				
		Strongly Agree	Agree	Neutral	Disagree	Strongly Disagree
1.	The patient’s health outcomes would be improved when medications are monitored by a pharmacist as compared with other healthcare providers.	38.4, 38.8	47.8, 47.0	9.5, 9.5	2.3, 2.3	2.0, 2.4
		(35.6–42.1)	(43.7–50.3)	(7.7–11.6)	(1.5–3.5)	(1.5–3.7)
2.	Besides the processes of normal dispensing functions, reviewing patient’s medication profile and providing interventions are important as roles of pharmacists in preventing adverse effects.	49.2, 50.1	47.8, 46.5	1.4, 1.8	0.4, 0.4	1.1, 1.2
		(46.8–53.4)	(43.2–49.8)	(1.0–3.0)	(0.2–1.2)	(0.7–2.2)
3.	Creating a personal medication record can help patients avoid medication-related issues.	46.3, 47.1	50.6, 49.1	1.9, 2.5	0.2, 0.1	1.1, 1.2
		(43.8–50.4)	(45.9–52.4)	(1.5–3.9)	(0.0–0.6)	(0.6–2.2)
4.	Medication-related action plans help healthcare professionals and patients identify medication-related issues and evaluate their solutions.	35.3, 35.7	60.4, 59.5	3.1, 3.5	0.2, 0.2	1.0, 1.1
		(32.6–38.9)	(56.2–62.8)	(2.4–5.0)	(0.0–1.0)	(0.6–2.1)
5.	Counseling is an intervention I will provide to improve the patient’s understanding of the treatment.	47.6, 48.2	48.6, 48.0	2.4, 2.2		1.4, 1.6
		(44.9–51.5)	(44.7–51.3)	(1.4–3.4)		(1.0–2.8)
6.	Consistent documentation and regular control visits are the essential components of MTM services.	32.7, 33.2	60.4, 59.1	6.0, 6.7	0.1, 0.0	0.8, 0.9
		(30.2–36.4)	(55.9–62.3)	(5.2–8.6)	(0.0–0.3)	(0.4–1.8)
7.	By considering the five core elements of MTM: medication therapy review, personal medication record, medication-related action plan, intervention or referral, and documentation and follow-up, do you agree that MTM services are valuable?	47.1, 48.3	48.9, 47.5	2.9, 3.0	0.1, 0.1	1.1, 1.1
		(45.0–51.5)	(44.3–50.8)	(2.0–4.4)	(0.0–1.0)	(0.6–2.0)
8.	By applying MTM services, patients would receive adequate and beneficial information about their chronic disease(s) and medication therapies from their providers.	39.8, 40.6	56.5, 55.3	2.7, 3.1	0.1, 0.1	0.9, 1.0
		(37.4–43.9)	(52.0–58.6)	(2.1–4.5)	(0.0–0.6)	(0.5–1.9)
9.	Providing MTM services is a unique opportunity for pharmacists to participate in patient care in a broader spectrum.	38.3, 40.0	56.3, 54.3	4.3, 4.5	0.2, 0.1	1.0, 1.1
		(36.8–43.3)	(50.9–57.6)	(3.3–6.1)	(0.0–0.3)	(0.6–2.2)
10.	Applying MTM services requires more knowledge than basic information on pharmacy practice.	42.3, 43.2	50.7, 50.0	4.9, 4.7	1.1, 1.2	1.0, 1.0
		(40.0–46.5)	(46.7–53.3)	(3.4–6.3)	(0.6–2.1)	(0.5–2.0)

1 CI, confidence interval.

The median of the total attitude score was 43 (min. = 10, max. = 50) out of 50. The median split resulted in 53.6 and 46.4% of pharmacists with positive and negative attitudes. Despite lowering the cutoff value by 10%, the proportion of those with positive attitudes remained higher than those with negative attitudes. However, increasing by 10% resulted in different findings, with a higher number of those with negative attitudes than positive attitudes (Figure 2). Increasing by 10% made the cutoff point too high (94.6% of the maximum score), causing the frequency to change dramatically.

### 3.4. Pharmacists’ practice toward MTM

The respondents generally engage in practices that support MTM implementation. They reported being able to cooperate with other healthcare workers in caring for patients (85.06%) and specifically review the treatment of patients identified as having drug-related problems (73.28%). Fewer practices were related to personal medication records (45.7%) and medication-related action plans (40.55%). Table 4 shows the proportion of “Yes” answers to daily practices that could support MTM implementation.

**Table 4:** Pharmacists’ practice toward MTM.

No.	Items	Correct Answers		
		Unweighted		Weighted
		*n*	%	% (95% CI^1^)
1.	Do you use the patient’s medical records in communicating and working with other health workers to achieve optimal treatment goals?	781	69.0	69.2 (66.1–72.2)
2.	Do you specifically review the treatment of patients identified as having drug-related problems?	823	72.7	73.3 (70.3–76.1)
3.	Do you create and provide a personal medication record for patients?	500	44.2	45.7 (42.4–49.0)
4.	Do you provide a record of the action plan for the patient so they can observe the progress in their treatment?	455	40.2	40.6 (37.3–43.9)
5.	Do you design and implement strategies to address or prevent medication-related problems?	686	60.6	60.8 (57.5–64.0)
6.	Are you able to cooperate with other health professionals in caring for patients?	955	84.4	85.1 (82.7–87.2)
7.	To evaluate the progress of your patient’s treatment, do you document the services and interventions provided?	685	60.5	62.1 (58.9–65.2)
8.	Do you evaluate the progress of the patient’s treatment?	678	59.9	60.7 (57.5–63.8)

1 CI, confidence interval.

In addition, we asked questions to gather additional information regarding MTM practices. Accordingly, more than 85% of respondents were willing to become MTM service providers. However, less than 50% believe that they will have enough time to implement the service. Approximately 70% of the pharmacists reported having enough time to provide counseling, whereas only 34.02% had a private area. More than 90% of the respondents were interested in attending training on MTM, and 59% preferred a face-to-face workshop.

A sub-analysis was conducted to determine the percentage of supporting information between pharmacists who work at inpatient and outpatient CHCs. Accordingly, pharmacists who worked in inpatient (48.9%) and outpatient (45.3%) settings thought that they would have enough time to implement MTM services. Only 29.2% of inpatient pharmacists and 37.3% of outpatient pharmacists had private areas for counseling. Over 60% of pharmacists in both practice settings accessed the “Guidelines for Basic Medication in Community Health Center” (online or hard copy) to support the review of patient treatment. Table 5 presents the proportion of answers to questions about additional information regarding MTM practices.

**Table 5:** Additional information regarding MTM practices.

No.	Items	“Yes” Answers		
		Unweighted *n*		Weighted % (95% CI^1^)
		Inpatient setting	Outpatient setting	Total
1.	If MTM will be implemented in the future, would you like to be an MTM provider?	365	626	88.8 (86.6–90.7)
2.	Do you think that you will have enough time to apply MTM services in the future?	186	301	46.8 (43.6–50.0)
3.	In your current practice, do you think that you spend enough time counseling your patients?	296	505	72.6 (69.6–75.4)
4.	Does the place where you work have a private counseling area?	136	279	34.0 (31.0–37.1)
5.	Do you access the “Guidelines for Basic Medication in the Community Health Center” (online or hard copy) to support the review of patient treatment?	253	447	61.9 (58.7–65.1)
6.	Do you access other guidelines and drug information resources (online or hard copies)?	353	611	84.8 (82.3–87.1)
7.	Do you think that a lack of training can hinder the implementation of MTM?	402	687	96.6 (95.3–97.6)
8.	Are you interested in learning more information about providing an MTM service?	380	661	92.6 (90.7–94.1)
9.	If yes, which method do you prefer:			
	- Online education	127	241	33.6 (30.5–36.7)
	- Live workshops	253	420	59.0 (55.7–62.2)

1 CI, confidence interval.

The total practice scores ranged from 0 to 8 (out of 8). With a median of 5, 57.9% were positive and 42.1% were negative practice categories. As shown in Figure 2, only increasing the cutoff value of 10% changed the finding of this study (the positive category became 45.7% and the low category became 54.3%).

### 3.5. Factors associated with pharmacists’ KAP

Table 6 shows the associations between pharmacists’ characteristics and KAP from the bivariate analyses, and Table 7 presents the results of the multivariable regressions. Gender, province, and experience in providing MTM services were all significantly associated with knowledge of MTM (*p* < 0.05) in both the bivariate and multivariable analyses. As shown in Table 7, men were 0.67 times less likely to have high knowledge than women. Furthermore, those without experience in MTM services provision were 0.65 times less likely to have high knowledge than those with MTM experience. Of the province factor, pharmacists in 12 provinces were 2.71–7.16 times more likely to have high knowledge than pharmacists in Southeast Sulawesi.

**Table 6:** Bivariate analysis results for the association between respondents’ characteristics and knowledge, attitude, and practices.

Characteristics	Knowledge		*p*-value	Attitude		*p*-value	Practice		*p*-value
	Low n^1^ (%^2^)	High n^1^ (%^2^)		Negative *n*^1^ (%^2^)	Positive *n*^1^ (%^2^)		Negative *n*^1^ (%^2^)	Positive *n*^1^ (%^2^)	
Gender									
Men	65 (6.9%)	180 (14.7%)	0.005*	98 (8.3%)	147 (13.3%)	0.005*	102 (8.5%)	143 (13.1%)	0.318
Women	214 (18.2%)	673 (60.2%)		439 (38.1%)	448 (40.3%)		395 (33.7%)	492 (44.8%)	
Age (years)									
20–30	146 (12.4%)	432 (39.1%)	0.781	275 (23.9%)	303 (27.6%)	0.938	261 (21.6%)	317 (29.9%)	0.642
31–40	114 (11.0%)	354 (30.5%)		224 (19.4%)	244 (22.1%)		203 (17.8%)	265 (23.7%)	
41–50	16 (1.4%)	58 (4.7%)		33 (2.7%)	41 (3.4%)		30 (2.5%)	44 (3.7%)	
> 50	3 (0.2%)	9 (0.6%)		5 (0.3%)	7 (0.5%)		3 (0.2%)	9 (0.6%)	
Educational background									
Pharmacist professional	268 (23.9%)	821 (72.0%)	0.612	522 (45.1%)	567 (50.8%)	0.055	484 (41%)	30 (2.9%)	0.052
Master	11 (1.2%)	32 (2.9%)		15 (1.3%)	28 (2.8%)		13 (1.2%)	0	
Doctoral	0	0		0	0		0		
Practice setting									
Inpatient	107 (10.7%)	314 (30.3%)	0.561	182 (16.6%)	239 (24.4%)	< 0.001*	190 (17.7%)	231 (23.3%)	0.583
Outpatient	172 (14.4%)	539 (44.6%)		355 (29.8%)	356 (29.2%)		307 (24.5%)	404 (34.5%)	
Province									
Aceh	9 (1.1%)	33 (4.0%)	0.001*	21 (2.5%)	21 (2.5%)	0.006*	15 (1.8%)	27 (3.2%)	0.029*
North Sumatra	17 (1.2%)	52 (3.9%)		30 (2.2%)	39 (2.9%)		26 (1.9%)	43 (3.2%)	
West Sumatra	7 (0.4%)	39 (2.5%)		19 (1.2%)	27 (1.7%)		24 (1.5%)	22 (1.4%)	
Riau	6 (0.7%)	18 (2.2%)		7 (0.9%)	17 (2.1%)		6 (0.7%)	18 (2.2%)	
Jambi	9 (0.9%)	15 (1.5%)		7 (0.7%)	17 (1.7%)		6 (0.9%)	15 (1.5%)	
South Sumatra	3 (0.4%)	24 (3.7%)		12 (1.9%)	15 (2.3%)		10 (1.5%)	17 (2.6%)	
Bengkulu	4 (0.5%)	6 (0.9%)		3 (0.4%)	7 (1.0%)		5 (0.7%)	5 (0.7%)	
Lampung	5 (0.4%)	33 (2.3%)		17 (1.2%)	21 (1.4%)		17 (1.1%)	21 (1.4%)	
Bangka Belitung	4 (0.4%)	4 (0.4%)		6 (0.7%)	2 (0.3%)		5 (0.6%)	3 (0.4%)	
Riau Islands	3 (0.3%)	9 (0.7%)		5 (0.4%)	7 (0.5%)		6 (0.4%)	6 (0.4%)	
Jakarta	6 (1.0%)	21 (3.4%)		17 (2.7%)	10 (1.6%)		12 (1.9%)	15 (2.4%)	
West Java	71 (2.7%)	217 (8.2%)		141 (5.4%)	147 (5.6%)		132 (5.0%)	156 (5.9%)	
Central Java	30 (2.3%)	100 (7.5%)		65 (5.0%)	65 (5.0%)		61 (4.6%)	69 (5.2%)	
Yogyakarta	4 (0.7%)	5 (1.0%)		6 (1.2%)	3 (0.5%)		2 (0.4%)	7 (1.3%)	
East Java	13 (1.9%)	51 (7.3%)		27 (3.9%)	37 (5.3%)		20 (2.8%)	44 (6.3%)	
Banten	13 (0.8%)	30 (1.8%)		24 (1.4%)	19 (1.2%)		15 (0.9%)	28 (1.7%)	
Bali	7 (0.4%)	15 (0.9%)		13 (0.8%)	9 (0.5%)		15 (0.9%)	7 (0.4%)	
West Nusa Tenggara	2 (0.3%)	18 (2.6%)		10 (1.4%)	10 (1.4%)		6 (0.9%)	14 (2.0%)	
East Nusa Tenggara	8 (1.3%)	16 (2.7%)		9 (1.5%)	15 (2.6%)		12 (2.0%)	12 (2.0%)	
West Kalimantan	11 (0.8%)	24 (1.7%)		13 (0.9%)	22 (1.6%)		21 (1.5%)	14 (1.0%)	
Central Kalimantan	8 (0.7%)	16 (1.4%)		14 (1.2%)	10 (0.9%)		12 (1.1%)	12 (1.1%)	
South Kalimantan	5 (0.7%)	16 (2.3%)		10 (1.4%)	11 (1.6%)		11 (1.6%)	10 (1.4%)	
East Kalimantan	6 (0.4%)	30 (2.2%)		22 (1.6%)	14 (1.1%)		18 (1.3%)	18 (1.3%)	
North Kalimantan	5 (0.3%)	9 (0.4%)		7 (0.4%)	7 (0.4%)		6 (0.4%)	8 (0.4%)	
North Sulawesi	4 (0.6%)	8 (1.2%)		6 (0.9%)	6 (0.9%)		8 (1.2%)	4 (0.6%)	
Central Sulawesi	4 (1.0%)	12 (1.9%)		9 (1.4%)	9 (1.4%)		7 (1.1%)	11 (1.8%)	
South Sulawesi	6 (1.1%)	25 (4.7%)		15 (2.8%)	16 (3.0%)		11 (2.1%)	20 (3.8%)	
Southeast Sulawesi	7 (1.6%)	7 (1.6%)		2 (0.4%)	12 (2.8%)		5 (1.1%)	9 (2.1%)	
The Community Health Center provides the drug for the government program									
No	10 (0.6%)	27 (2.3%)	0.6	18 (1.7%)	19 (1.3%)	0.259	11 (0.8%)	26 (2.2%)	0.06
Yes	269 (24.5%)	826 (72.6%)		519 (44.7%)	576 (52.3%)		486 (41.4%)	609 (55.7%)	
Years of practice									
0–10	250 (22.4%)	767 (67.8%)	0.783	479 (41.2%)	538 (48.9%)	0.248	438 (37.1%)	579 (53.1%)	0.019*
11–20	25 (2.5%)	74 (6.3%)		53 (4.8%)	46 (4%)		54 (4.7%)	45 (4%)	
21–30	3 (0.1%)	8 (0.5%)		4 (0.2%)	7 (0.4%)		5 (0.4%)	6 (0.3%)	
> 30	1 (0.1%)	4 (0.4%)		1 (0.2%)	4 (0.3%)		0	5 (0.5%)	
Have ever provided MTM services									
No	162 (14.6%)	409 (35.6%)	0.002*	289 (24.8%)	282 (25.4%)	0.036*	347 (29.6%)	224 (20.6%)	<0.001*
Yes	117 (10.5%)	444 (39.3%)		248 (21.5%)	313 (28.2%)		150 (12.5%)	411 (37.3%)	

1 Unweighted *n*.

2 Weighted %.

* Significant at *p* ≤ 0.05.

**Table 7:** Multivariate analysis results for predictors of pharmacists’ knowledge, attitude, and practices.

Characteristics	Knowledge^1^			Attitude^2^			Practice^2^		
	SE^3^	*p*-value	OR^4^ (95% CI^5^)	SE^3^	*p*-value	OR^4^ (95% CI^5^)	SE^3^	*p*-value	OR^4^ (95% CI^5^)
Gender									
Men	0.17	0.019*	0.67 (0.48–0.94)	0.16	0.016*	1.46 (1.07–1.99)	0.17	0.27	1.2 (0.87–1.67)
Women (Ref.)			1			1			1
Age (years)									
20–30	0.92	0.673	1.48 (0.24–9.01)	0.83	0.947	0.95 (0.19–4.79)	0.88	0.182	0.31 (0.05–1.74)
31–40	0.92	0.64	1.54 (0.25–9.41)	0.83	0.976	1.02 (0.2–5.18)	0.88	0.241	0.36 (0.06–2)
41–50	0.95	0.509	1.87 (0.29–12.07)	0.85	0.775	1.27 (0.24–6.71)	0.9	0.392	0.46 (0.08–2.7)
> 50 (Ref.)			1			1			1
Educational background									
Pharmacist professional	0.37	0.647	1.18 (0.58–2.44)	0.34	0.125	0.59 (0.3–1.16)	0.37	0.077	0.52 (0.25–1.07)
Master (Ref.)			1			1			1
Practice setting									
Inpatient	0.15	0.14	0.8 (0.59–1.08)	0.14	0.007*	1.45 (1.11–1.89)	0.15	0.114	0.8 (0.6–1.06)
Outpatient (Ref.)			1			1			1
Province									
Aceh	0.47	0.011*	3.33 (1.33–8.39)	0.55	0.001*	0.17 (0.06–0.5)	0.48	0.732	0.85 (0.33–2.16)
North Sumatra	0.46	0.06	2.38 (0.97–5.85)	0.55	0.011*	0.25 (0.08–0.73)	0.47	0.39	0.67 (0.26–1.68)
West Sumatra	0.6	0.005^*^	5.32 (1.65–17.1)	0.6	0.027*	0.27 (0.08–0.86)	0.53	0.184	0.49 (0.17–1.4)
Riau	0.52	0.058	2.7 (0.97–7.54)	0.61	0.13	0.4 (0.12–1.31)	0.56	0.492	1.47 (0.49–4.39)
Jambi	0.53	0.44	1.5 (0.54–4.21)	0.64	0.189	0.43 (0.12–1.51)	0.56	0.954	0.97 (0.32–2.93)
South Sumatra	0.58	0.001*	7.16 (2.32–22.13)	0.56	0.007*	0.22 (0.07–0.67)	0.49	0.872	0.92 (0.35–2.42)
Bengkulu	0.62	0.903	1.08 (0.32–3.63)	0.73	0.254	0.44 (0.1–1.81)	0.64	0.071	0.32 (0.09–1.1)
Lampung	0.64	0.005*	6.03 (1.71–21.24)	0.61	0.007*	0.19 (0.06–0.63)	0.55	0.175	0.48 (0.16–1.39)
Bangka Belitung	0.71	0.861	0.88 (0.22–3.55)	0.86	0.001*	0.06 (0.01–0.33)	0.77	0.075	0.25 (0.06–1.15)
Riau Islands	0.79	0.185	2.86 (0.6–13.55)	0.79	0.104	0.28 (0.06–1.3)	0.75	0.611	0.68 (0.16–2.96)
Jakarta	0.49	0.021*	3.09 (1.18–8.09)	0.57	< 0.001*	0.12 (0.04–0.37)	0.49	0.419	0.67 (0.26–1.75)
West Java	0.4	0.012*	2.71 (1.24–5.92)	0.51	0.001*	0.19 (0.07–0.51)	0.42	0.136	0.54 (0.24–1.22)
Central Java	0.41	0.007*	3.04 (1.36–6.77)	0.51	0.001*	0.18 (0.07–0.49)	0.42	0.217	0.59 (0.26–1.36)
Yogyakarta	0.59	0.912	0.94 (0.3–2.96)	0.69	< 0.001*	0.09 (0.02–0.34)	0.7	0.73	1.27 (0.32–5.04)
East Java	0.42	0.001*	3.89 (1.71–8.83)	0.52	0.003*	0.22 (0.08–0.61)	0.44	0.666	1.21 (0.51–2.83)
Banten	0.53	0.189	2.01 (0.71–5.69)	0.61	0.002*	0.15 (0.05–0.5)	0.56	0.913	1.06 (0.36–3.17)
Bali	0.66	0.275	2.05 (0.57–7.41)	0.71	0.006*	0.14 (0.03–0.56)	0.7	0.057	0.26 (0.07–1.04)
West Nusa Tenggara	0.69	0.002*	8.2 (2.14–31.4)	0.6	0.001*	0.15 (0.04–0.47)	0.55	0.919	0.95 (0.32–2.8)
East Nusa Tenggara	0.47	0.146	1.97 (0.79–4.9)	0.57	0.023*	0.27 (0.09–0.83)	0.49	0.25	0.57 (0.22–1.49)
West Kalimantan	0.53	0.123	2.28 (0.8–6.49)	0.62	0.046*	0.29 (0.09–0.98)	0.55	0.148	0.45 (0.15–1.33)
Central Kalimantan	0.55	0.316	1.74 (0.59–5.11)	0.63	0.001*	0.13 (0.04–0.46)	0.57	0.187	0.47 (0.15–1.44)
South Kalimantan	0.53	0.03*	3.19 (1.12–9.07)	0.59	0.006*	0.19 (0.06–0.62)	0.53	0.133	0.45 (0.16–1.27)
East Kalimantan	0.6	0.015*	4.33 (1.34–14.05)	0.61	< 0.001^*^	0.11 (0.03–0.36)	0.54	0.129	0.44 (0.15–1.27)
North Kalimantan	0.8	0.54	1.63 (0.34–7.8)	0.84	0.042*	0.18 (0.03–0.94)	0.83	0.689	0.72 (0.14–3.64)
North Sulawesi	0.59	0.282	1.88 (0.6–5.97)	0.66	0.007*	0.17 (0.05–0.62)	0.63	0.036*	0.27 (0.08–0.92)
Central Sulawesi	0.51	0.259	1.77 (0.66–4.79)	0.59	0.003*	0.17 (0.05–0.54)	0.54	0.618	0.76 (0.26–2.21)
South Sulawesi	0.46	0.004*	3.8 (1.54–9.37)	0.54	0.002*	0.18 (0.06–0.53)	0.46	0.858	1.09 (0.44–2.68)
Southeast Sulawesi (Ref.)			1			1			1
The Community Health Center provides the drug for the government program									
No	0.44	0.885	1.07 (0.45–2.54)	0.54	0.002*	0.18 (0.06–0.54)	0.43	0.015*	2.87 (1.23–6.69)
Yes (Ref.)			1			1			1
Years of practice									
0–10	1.18	0.838	0.79 (0.08–7.89)	1.02	0.586	0.57 (0.08–4.26)	0.26	0.015*	1.89 (1.13–3.16)
11–20	1.2	0.667	0.6 (0.06–6.24)	1.04	0.35	0.38 (0.05–2.91)			16
21–30	1.44	0.972	0.95 (0.06–15.87)	1.25	0.889	1.19 (0.1–13.87)			
> 30 (Ref.)			1			1			
Have ever provided MTMv services									
No	0.15	0.003*	0.65 (0.48–0.86)	0.13	0.033*	0.76 (0.6–0.98)	0.14	<0.001*	0.23 (0.17–0.29)
Yes (Ref.)			1			1			1

1 Using multivariable ordinal regression.

2 Using multivariable logistic regression.

3 SE, standard error.

4 OR, odds ratio.

5 CI, confidence interval.

^6^The reference variable of years of practice in regression for the predictors of practice was >10 years.

* Significant at *p* ≤ 0.05.

Regarding attitudes, both analyses show that gender, practice settings (inpatient or outpatient), province, and experience in providing MTM services were predictors of pharmacists’ attitudes toward MTM. Men were 1.46 times more likely to have positive attitudes than women. In addition, pharmacists in inpatient settings tended to have positive attitudes 1.45 times more than those in outpatient settings. Pharmacists without experience in MTM provision were 0.76 times less likely to have a positive attitude than those with experience. Moreover, 23 provinces were significantly associated with less positive attitudes than the reference (odds ratio [OR], 0.06–0.29).

The bivariate and multivariable analyses identified three predictors of pharmacists’ practice toward MTM: province (North Sulawesi: OR = 0.27; 0.08–0.92), years of practice (OR = 1.89; 1.13–3.16), and experience providing MTM services (OR = 0.23; 0.17–0.29). Although insignificant in the bivariate analysis, the logistic regression found that the provision of the drug of the government program was significantly associated with positive practices toward MTM (OR = 2.87; 1.23–6.69).

### 3.6. Pharmacists’ perceptions of barriers and facilitators regarding MTM provision in the future

**Figure 3:**
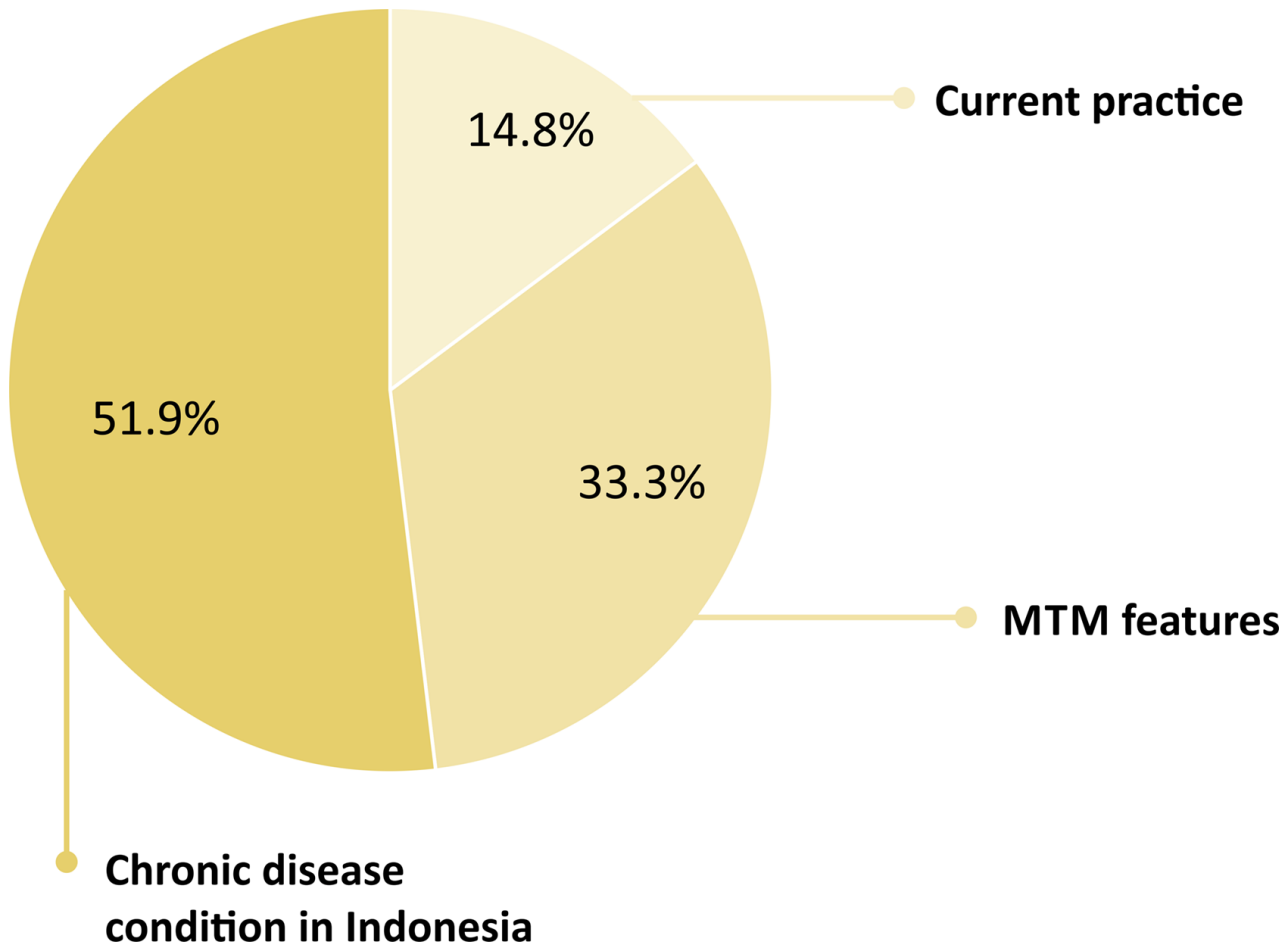
Categories of the medication therapy management facilitators perceived by respondents.

Regarding facilitators, three categories were identified: chronic disease conditions in Indonesia, MTM features, and current practices which support MTM implementation. With the increasing prevalence of chronic diseases in Indonesia and the large population of patients with high-risk status, respondents thought that MTM services were promising. Moreover, respondents believed that the five core elements could facilitate achieving the therapeutic goals. On the contrary, many current practices — for example, medication reviews, counseling, and visits — could support the adoption of MTM. Figure 3 displays the proportion of categories of MTM facilitators perceived by respondents.

**Figure 4:**
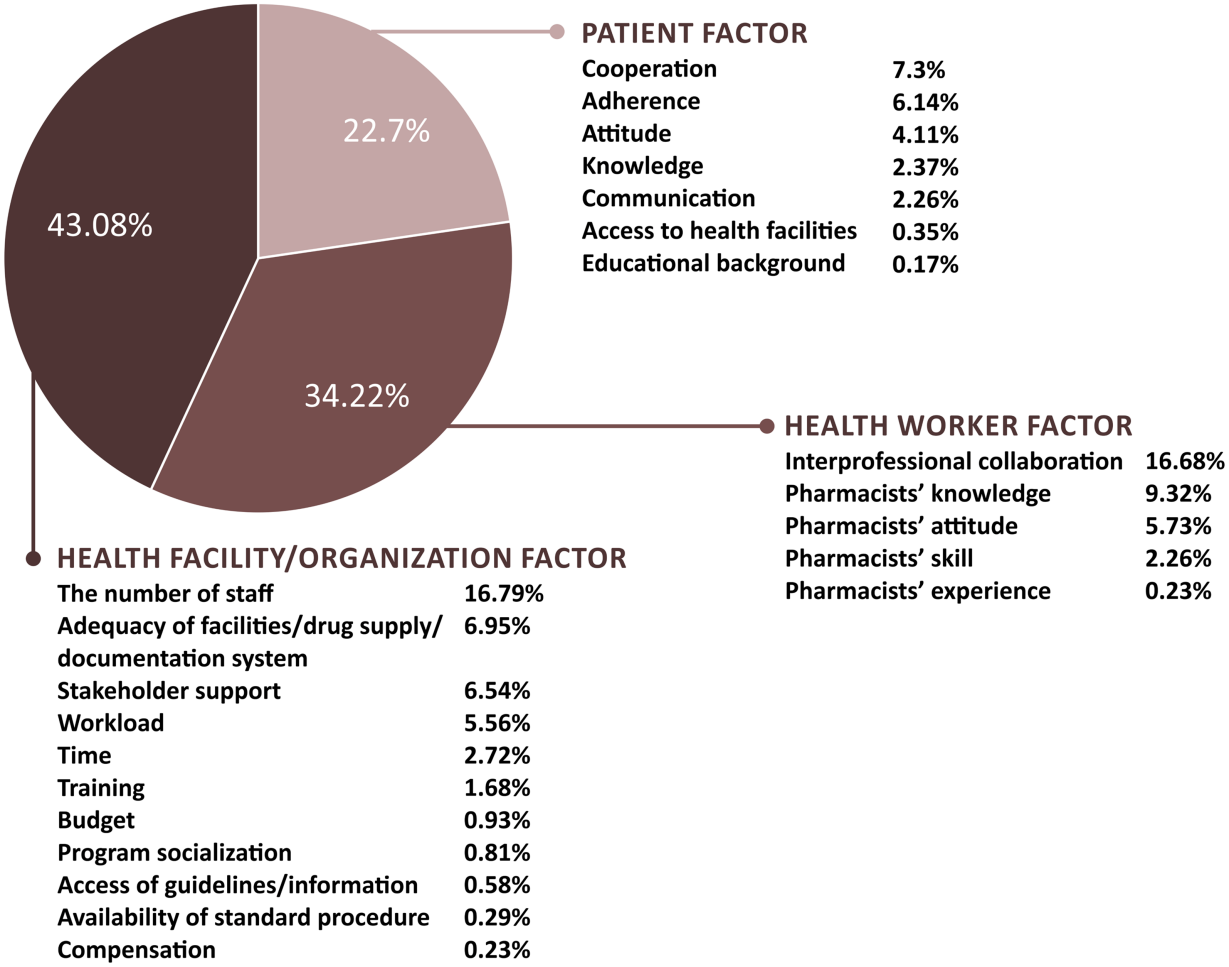
Categories of medication therapy management barriers perceived by respondents.

Concerning the barriers to MTM implementation, limited staff, lack of interprofessional collaboration, lack of pharmacist knowledge, low patient cooperation, lack of facilities/drug supply/documentation systems, lack of stakeholder support, and low patient compliance were the most perceived barriers. In addition, 23 codes were identified, and they were grouped into 10 categories and then into the three factors: health facility/organization, health worker, and patient factors (Figure 4). Most of these barriers were related to the health facility/organization factor (42.73%), followed by the health worker factor (34.99%) and patient factor (22.29%).

**Figure 5:**
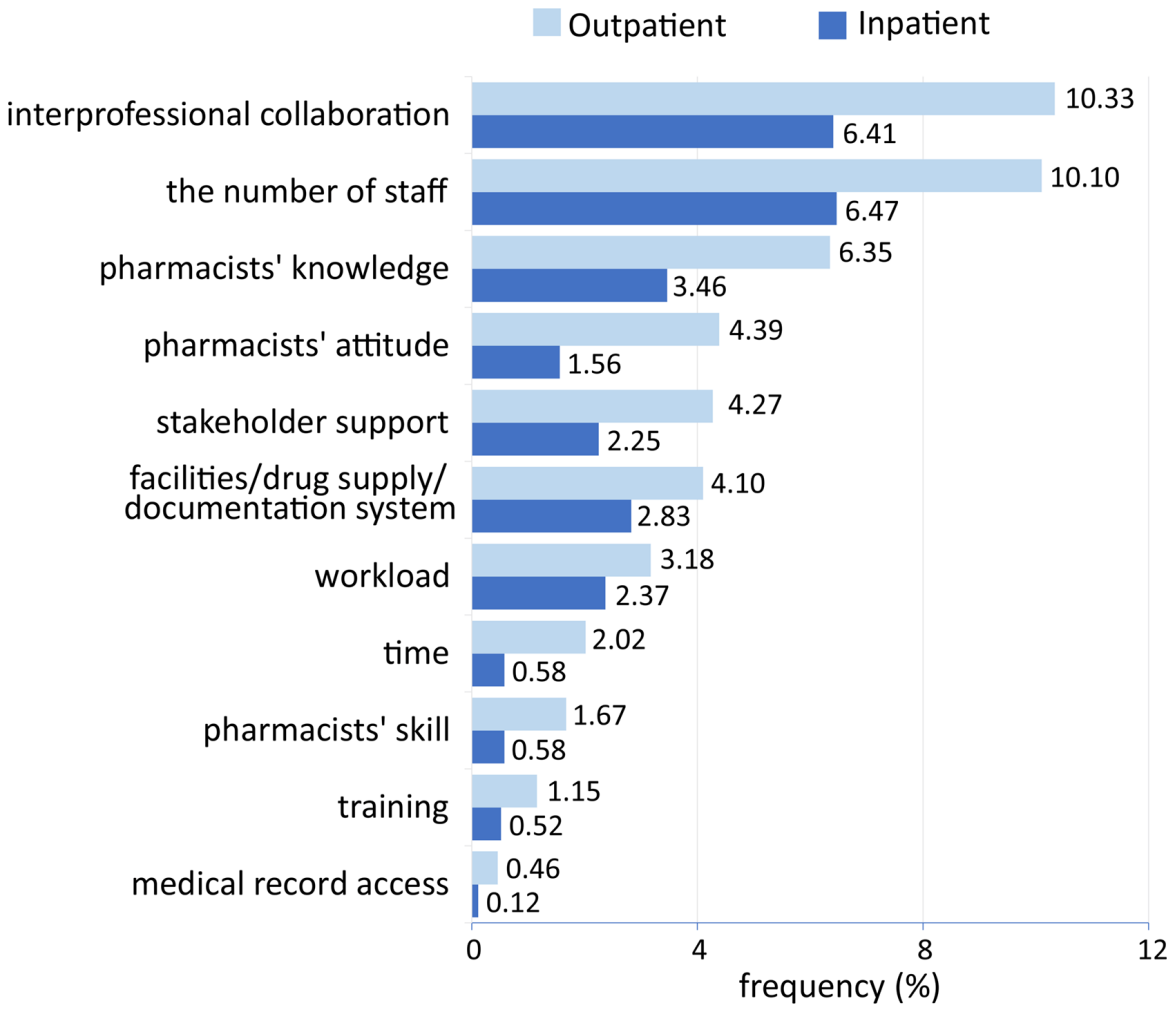
Barriers perceived by pharmacists based on the practice settings.

**Figure 6:**
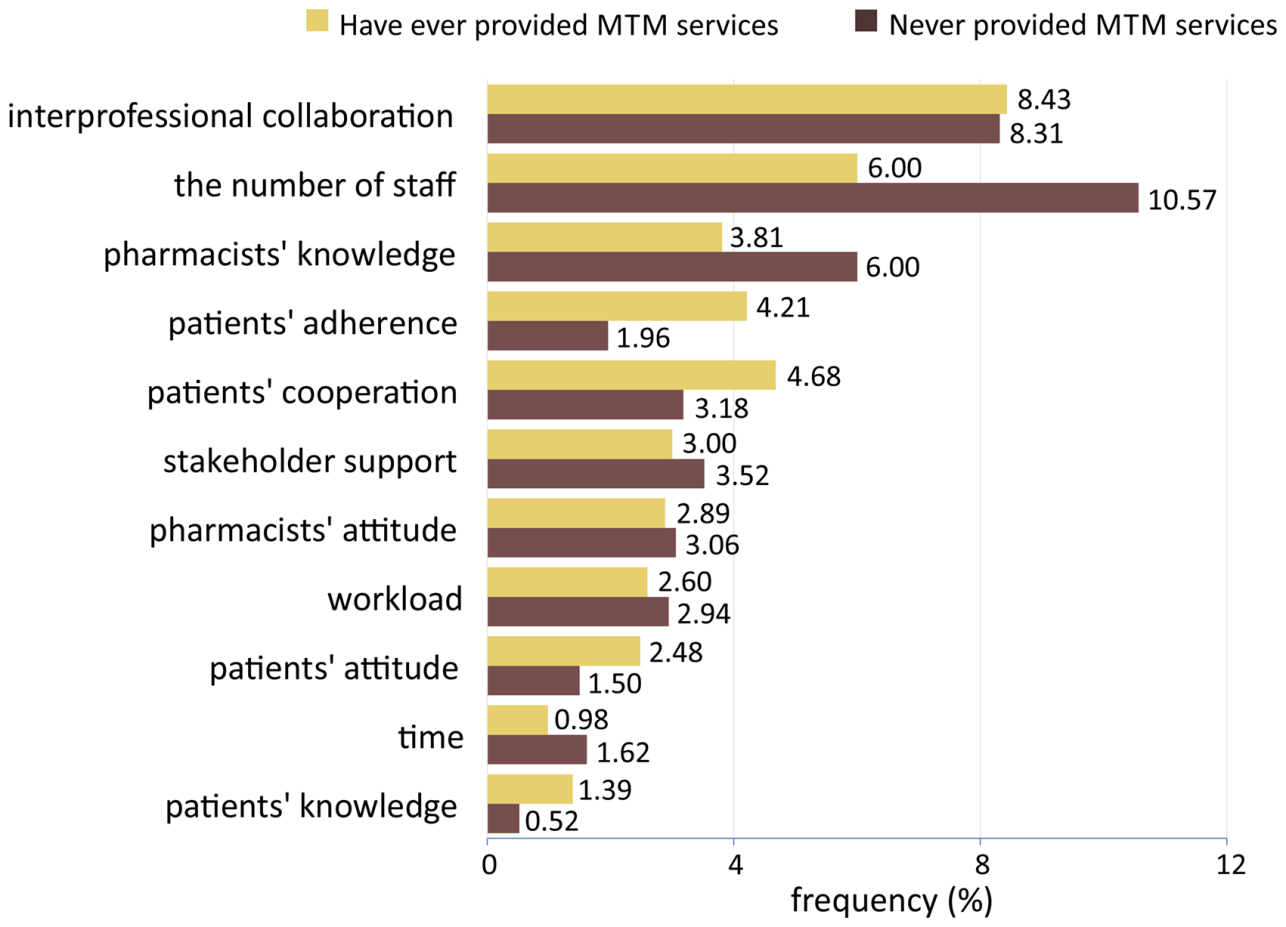
Barriers perceived by pharmacists based on their experience in providing medication therapy management services.

Respondents implied that barriers categorized into the health worker factor were highly related to pharmacists’ KAP. Knowledge of medication therapy and MTM services; attitudes toward MTM, patient-oriented services, and changes in the pharmacy practice; skills in providing pharmaceutical services, communicating with patients, and collaborating with other professionals became their concerns toward MTM implementation. Despite organizational and patient factors differences, pharmacists in inpatient and outpatient settings and those who have provided and have not provided MTM services perceived similar barriers related to their KAP of MTM (Figures 5, 6).

## 4. Discussion

Understanding the level of KAP of pharmacists as key providers of MTM services is crucial to the program implementation success. This study shows that approximately 75% of pharmacists in CHCs had a high level of knowledge about MTM. Nonetheless, less than 58% of pharmacists had a positive attitude and practice toward MTM.

Regarding knowledge, most respondents understood the scope of therapies covered in MTM and could characterize all five core elements, especially regarding documentation and personal medication records. However, respondents appeared to need more understanding of the comprehensive and targeted medication therapy review, medication-related action plans, and interventions. Medication therapy review is not a new practice for pharmacists in CHCs, as stated in the Technical Guidelines for Pharmaceutical Service Standards at Community Health Center (33). Nevertheless, the term comprehensive and targeted review might not be familiar. A study in Jordan has reported that pharmacists were knowledgeable about medication therapy reviews as MTM elements, but they did not report whether pharmacists were knowledgeable about the two types of reviews involved in MTM (28).

In addition, less than 58% of the respondents showed a positive attitude toward MTM services. Respondents generally agreed on pharmacists’ role in all five core elements of the MTM. Respondents also agreed that the provision of MTM services is a unique opportunity to expand the role of pharmacists in patient care. KAP studies on MTM in other countries, such as Saudi Arabia, Malaysia, and Jordan, showed similar results (1, 18, 28). However, we were concerned about the difference between the proportion at the level of knowledge and the proportion of the attitude regarding MTM. The study respondents showed an almost balanced proportion of positive and negative attitudes. This indicates that some respondents were uncertain about MTM implementation, even though they already understood the concept (34). This is in line with the finding of the study by Ahamad and Ariffin (35) on KAP regarding sustainable consumption among students in Malaysia. In certain concepts, experience is more influential than textual knowledge toward attitude (35).

In this study, we measured practices toward MTM based on pharmacists’ daily activities that support MTM implementation. Most pharmacists reported positive practices toward MTM, including interprofessional collaboration in interventions and referrals and a specific review regarding the therapy of patients with drug-related problems. However, there was an almost equal distribution between the positive and negative practice categories. Respondents reported not being accustomed to designing and implementing strategies to address or prevent treatment-related issues, documenting services, or evaluating medication progress. It is in line with the study by Wijaya et al. (26) that found pharmacists at CHCs were lack of skill to manage patient compliance and review patients’ therapy (26).

Further information about practices could provide more insights, including pharmacists’ willingness to provide MTM services (88%), their interest in attending MTM training (92.55%), and their preference for face-to-face training sessions (58%). The rates of willingness to become a provider compared with interest in training indicated the need for educational programs to improve pharmacists’ readiness. The results were similar to those of a study of community pharmacists in Lebanon (36). In addition, the proportion of respondents who felt that they had enough time to implement MTM was higher among pharmacists working in inpatients than in outpatient settings. It may be related to the number of staff and workload at inpatient CHCs (37). Conversely, fewer pharmacists working in inpatient settings reported the availability of private counseling areas/rooms. The counseling session will be conducted in an integrated room if a private room is not available in the CHC (37).

In terms of attitudes, men tend to have a positive attitude toward MTM compared with women. There may be a burnout tendency in women due to the increased workload that they perceive when providing MTM services (38). Respondents working in inpatient settings also tend to have a more positive attitude toward MTM, which could be related to their familiarity with the services. Pharmacists in these settings routinely performed medication reviews, therapy evaluations, and ward visits (33). Similarly, experience in providing MTM services becomes a factor associated with pharmacists’ KAP. A systematic review suggests that factors increasing direct involvement in patient-oriented or MTM services can improve the understanding of MTM (10).

The study found that some provinces had significant associations, not only with high levels of knowledge but also with more negative attitudes and practices toward MTM. It might be related to the regional policies of health organizations/facilities, especially those directly related to pharmacists’ KAP, such as policy on training, the scope of responsibilities, and guidelines (39). In addition, pharmacists with ≤10 years of practice had more positive practices toward MTM. Similarly, Athiyah et al. (40) reported lower practice scores for pharmacists working for more than10 years. It might be related to the tendency to feel exhausted and burned out from prolonged work (40). Moreover, the provision of the drug of the government program was associated with MTM practice. Although not significant (*p* = 0.060) if considered alone in the bivariate analysis, this factor became significant (*p* = 0.015) when simultaneously analyzed with other variables. It might occur due to mediation (collinearity) or moderation (41).

The findings of the exploratory study gave some insights into the facilitators and barriers to MTM implementation perceived by the primary providers of MTM. Chronic disease conditions, MTM features, and current practices were facilitators of MTM. On the contrary, the lack of staff, collaboration between professions, pharmacist knowledge, patient cooperation, adequacy of facilities/drug supply/documentation systems, stakeholder support, and patient compliance could hinder MTM implementation. Generally, the health facility/organization factor became the main barrier for pharmacists. Pharmacists in the studies in other countries perceived similar barriers to MTM, including the lack of time, staffing, compensation, and training (10). Fewer identified facilitators indicated that pharmacists appeared to emphasize barriers and overlook facilitators. A study on maternal health evidence products in low-and middle-income countries suggested that discovering facilitators is more challenging, especially during the initial stages of program implementation (42).

According to the categories in the health worker factor, pharmacists were worried about unfavorable knowledge, attitudes, and skills that might impede the provision of MTM. The study in Jordan raised similar concerns about the challenges in implementing pharmacists-provided MTM, including those related to pharmacists’ skills in communicating with patients and collaborating with other health workers (28). Since medication management becomes a shared professional responsibility to ensure optimal drug safety and patient care (43), health workers should collaborate well. Studies have proposed designing a particular system and process to facilitate effective interaction and communication between pharmacists, doctors, and other health workers (43, 44). Thus, the mastery of the medical coding of diseases (International Classification of Diseases), particularly the 144 illnesses covered by BPJS, is one of the additional competencies for pharmacists in Indonesia to facilitate effective communication with doctors.

Based on the practice settings, pharmacists working in inpatient settings perceived fewer barriers related to organizational factors. It might correlate with the government regulation about additional resources to improve inpatient capabilities (37). Furthermore, fewer inpatient pharmacists perceived pharmacists’ competencies and collaboration as potential barriers. Pharmacists in inpatient settings are more likely to improve their skills and interprofessional relationship because of their daily practices (37). Based on the MTM experience, pharmacists who have provided MTM services reported more barriers regarding professional collaboration and patient factors. On the contrary, pharmacists without MTM experience perceived more obstacles related to the health facility/organization factor. This is consistent with the results of a study conducted in the United States, which compared barriers between pharmacists who would and were providing MTM services. The study found that those who would provide MTM perceived the lack of staff and access to medical information, whereas those who currently provide MTM reported compensation (45).

To the best of our knowledge, this is the first thorough evaluation of KAP toward MTM and its predictors among pharmacists in CHCs. The results of this study demonstrated the need to improve pharmacists’ KAP regarding MTM. Information about the predictors of pharmacists’ KAP suggests that interventions should increase pharmacist involvement. The fact that pharmacists preferred face-to-face workshops (59.45%) to online education (32.51%) supports this suggestion. This study also proposes that training should improve collaboration between pharmacists and other health workers and enhance pharmacist communication with patients. This study is also the first national survey involving CHC pharmacists in 28 provinces. Weighting on province variables was intended to make the study results more accurately represent the population of interest. It is an effort to minimize bias caused by non-probability sampling techniques (32).

Furthermore, we demonstrated how pharmacists think about the contribution of organizations/health facilities to the success of MTM implementation. CHC managers must provide adequate staffing or review the workload and responsibilities of pharmacists. CHC managers could set formal steps to enhance health workers’ collaboration, such as communication protocols that integrate pharmacists’ access to medical records (5). In addition, it is essential to ensure that the facilities, drug supply, and documentation systems are adequate and that standard operating procedures, regulations, and program socialization are available.

We propose practical suggestions such as a socialization and education program regarding MTM services before the nationwide implementation. A lack of understanding, for example about comprehensive and targeted review, should be addressed through an educational intervention (26). In addition, training is important to improve pharmacists’ attitude and practice towards MTM. Previous studies in the United States showed that training is effective in improving essential knowledge and attitude for pharmacists in providing patient-care services (46, 47). An intervention should also be designed to enhance pharmacists’ skill to design strategies in resolving drug-related problem, manage patients’ compliance, collaborate with other healthcare professionals, and communicate with patients. To ensure the effectiveness of the training program, this study suggest that pharmacists must be directly involved in the process.

This study has limitations. First, online survey methods are prone to self-selection bias. However, the widespread use of the internet, standardized competence of pharmacists, and participation credit from IAI could minimize the bias. Second, we could not calculate the response rate of the survey. Third, given the cross-sectional design, we could not infer a causal relationship among the factors of pharmacists’ KAP. Finally, the convenience sampling technique prevents generalizability. Nevertheless, participation from the 28 provinces and the application of weighting are considered to make the study results more representative.

Further research focusing on qualitative studies may complete our understanding regarding the KAP of MTM among pharmacists at CHCs. Probability sampling techniques and multivariable weighting (province, practice settings [inpatient and non-inpatient], and CHC accreditation) may be used to yield more representative results. Future studies may also focus on designing an educational intervention to improve pharmacists’ attitude and practice towards MTM. This study may provide valuable insight for program planners about considering health facility/organization factors in the strategy of MTM implementation.

## 5. Conclusion

Most respondents had high knowledge of MTM; however, only about half had positive attitudes and practices toward MTM. Gender, practice settings, province of CHCs, years of practice, and experience in MTM services were factors associated with KAP about MTM. Information about factors associated with the KAP level suggests that direct involvement is essential to improve pharmacists’ understanding and view of MTM. Respondents perceived that the chronic disease conditions in Indonesia, MTM service features, and current practices were facilitators of MTM provision. The lack of interprofessional collaboration, staff, pharmacist knowledge, patient cooperation, facilities/drug supply/documentation systems, stakeholder support, and patient compliance were the most common barriers to MTM implementation in the future. A training program is needed to improve the KAP about MTM and develop skills for collaborating with other healthcare professionals and communicating with patients. Qualitative research may further advance our understanding of KAP toward MTM among pharmacists in CHCs.

## Data availability statement

The raw data supporting the conclusions of this article will be made available by the authors, without undue reservation.

## Ethics statement

The studies involving human participants were reviewed and approved by Health Research Ethics Committee of Universitas Padjadjaran. The patients/participants provided their written informed consent to participate in this study.

## Author contributions

FR, SDA, and IMP: conceptualization, resources, and visualization. FR, SDA, WW, and IMP: methodology and writing—review and editing. FR: software, formal analysis, investigation, data curation, and writing—original draft preparation. SDA and IMP: validation. SDA, WW, and IMP: supervision. IMP: project administration and funding acquisition. All authors contributed to the article and approved the submitted version.

## Funding

This work was supported by Universitas Padjadjaran, Indonesia (grant number: 2203/UN6.3.1/PT.00/2022).

## Conflict of interest

The authors declare that the research was conducted in the absence of any commercial or financial relationships that could be construed as a potential conflict of interest.

## Publisher’s note

All claims expressed in this article are solely those of the authors and do not necessarily represent those of their affiliated organizations, or those of the publisher, the editors and the reviewers. Any product that may be evaluated in this article, or claim that may be made by its manufacturer, is not guaranteed or endorsed by the publisher.
