# Supplementary material for: Knowledge, attitude, and practice of medication therapy management: a national survey among pharmacists in Indonesia
Source: Front Public Health. 2023 Jul 17;11:1213520. doi: 10.3389/fpubh.2023.1213520 (PMC10388185; doi:10.3389/fpubh.2023.1213520)
Supplement: Supplementary file 2 [file Data_Sheet_2.PDF]

Table S2. The Matrix of Final Questionnaire.

Variabel	Indicator	Question/Statement
Indonesian Pharmacists Association ID Number	-	1. Indonesian Pharmacists Association ID Number:<br>……………………
Sociodemographic Characteristics	Gender	2. Gender:<br>☐ Male<br>☐ Female
	Age	3. Age (years):<br>☐ 20–30<br>☐ 31–40<br>☐ 41–50<br>☐ >50
	Educational background	4. Educational background:<br>☐ Pharmacist professional<br>☐ Master<br>☐ Doctoral
	Practice settings	5. Practice settings:<br>☐ Inpatient Community Health Center (CHC)<br>☐ Outpatient CHC
		6. Province of CHC:<br>…………………….
		7. Do the CHC where you work provide the drug of the government program?<br>☐ Yes<br>☐ No
	Years of practice	8. Years of practice:<br>☐ 0-10<br>☐ 11-20<br>☐ 21-30<br>☐ >30
	Experience in providing MTM service	Medication Therapy Management (MTM) is a service provided by health workers, including pharmacists, to ensure the best therapeutic outcomes for patients, including a review of the therapy to help patients better understand their health condition and manage their medications.<br><br>In Indonesia, MTM services were initiated by the government in collaboration with the Social Insurance Administration Organization (BPJS) Health in 2017. These services have been provided in Puskesmas, especially for chronic disease patients in the Referral Program (*PRB-berbasis MTM*).<br><br>9. Have you ever provided MTM services?<br>☐ Yes<br>☐ No

Knowledge	MTM beneficiaries/therapy coverage	1) Any patient who uses prescription and non-prescription medications, herbal products, or other dietary supplements could potentially benefit from MTM.<br>☐ True<br>☐ False<br>☐ Not sure
	Medication Therapy Review	2) Patients may receive a comprehensive medication therapy review once a year and a targeted medication therapy review if there are new medication-related issues.<br>☐ True<br>☐ False<br>☐ Not sure
	Personal Medication Record	3) In MTM service, the personal medication record serves as a tool for patients to manage their treatment based on the pharmacist's instructions.<br>☐ True<br>☐ False<br>☐ Not sure
	Medication-related Action Plan	4) Patients are actively involved in addressing medication-related problems by referring to the medication-related action plan records that pharmacists make.<br>☐ True<br>☐ False<br>☐ Not sure
	Intervention or Referral	5) Pharmacists' intervention to the medication therapy could be performed during MTM visits.<br>☐ True<br>☐ False<br>☐ Not sure
	Documentation and Follow-up	6) The documentation of MTM services includes recording the schedule of control visits, amount of time with patients, and feedback on health workers or patients.<br>☐ True<br>☐ False<br>☐ Not sure
Attitude	Pharmacists as the main provider of MTM	1. The patient's health outcomes would be improved when medications are monitored by a pharmacist as compared with other healthcare providers.<br>☐ 1 = strongly disagree<br>☐ 2 = disagree<br>☐ 3 = neutral<br>☐ 4 = agree<br>☐ 5 = strongly agree

	Pharmacists’ role in Medication Therapy Review	2. Besides the processes of normal dispensing functions, reviewing patient’s medication profile and providing interventions are important as roles of pharmacists in preventing adverse effects.<br>☐ 1 = strongly disagree<br>☐ 2 = disagree<br>☐ 3 = neutral<br>☐ 4 = agree<br>☐ 5 = strongly agree
	Pharmacists’ role in Personal Medication Record	3. Creating a personal medication record can help patients avoid medication-related issues.<br>☐ 1 = strongly disagree<br>☐ 2 = disagree<br>☐ 3 = neutral<br>☐ 4 = agree<br>☐ 5 = strongly agree
	Pharmacists’ role in Medication-related Action Plan	4. Creating a personal medication record can help patients avoid medication-related issues.<br>☐ 1 = strongly disagree<br>☐ 2 = disagree<br>☐ 3 = neutral<br>☐ 4 = agree<br>☐ 5 = strongly agree
	Pharmacists’ role in Intervention or Referral	5. Counseling is an intervention I will provide to improve the patient’s understanding of the treatment.<br>☐ 1 = strongly disagree<br>☐ 2 = disagree<br>☐ 3 = neutral<br>☐ 4 = agree<br>☐ 5 = strongly agree
	Pharmacists’ role in Documentation and Follow-up	6. Consistent documentation and regular control visits are the essential components of MTM services.<br>☐ 1 = strongly disagree<br>☐ 2 = disagree<br>☐ 3 = neutral<br>☐ 4 = agree<br>☐ 5 = strongly agree
	The benefit of the core elements of MTM	7. By considering the five core elements of MTM: medication therapy review, personal medication record, medication-related action plan, intervention or referral, and documentation and follow-up, do you agree that MTM services are valuable?<br>☐ 1 = strongly disagree<br>☐ 2 = disagree<br>☐ 3 = neutral<br>☐ 4 = agree<br>☐ 5 = strongly agree

	The benefit of MTM for patients' understanding of their disease and medication therapy	8. By applying MTM services, patients would receive adequate and beneficial information about their chronic disease(s) and medication therapies from their providers.<br>☐ 1 = strongly disagree<br>☐ 2 = disagree<br>☐ 3 = neutral<br>☐ 4 = agree<br>☐ 5 = strongly agree
	The expansion of the role of pharmacists through MTM implementation	9. Providing MTM services is a unique opportunity for pharmacists to participate in patient care in a broader spectrum.<br>☐ 1 = strongly disagree<br>☐ 2 = disagree<br>☐ 3 = neutral<br>☐ 4 = agree<br>☐ 5 = strongly agree
	Pharmacists' competency required to provide MTM	10. Applying MTM services requires more knowledge than basic information on pharmacy practice.<br>☐ 1 = strongly disagree<br>☐ 2 = disagree<br>☐ 3 = neutral<br>☐ 4 = agree<br>☐ 5 = strongly agree
Practice	Daily activities related to Medication Therapy Review	1. Do you use the patient's medical records in communicating and working with other health workers to achieve optimal treatment goals?<br>☐ Yes<br>☐ No<br>☐ Not sure
		2. Do you specifically review the treatment of patients identified as having drug-related problems?<br>☐ Yes<br>☐ No<br>☐ Not sure
	Daily activity related to Personal Medication Record	3. Do you create and provide a personal medication record for patients?<br>☐ Yes<br>☐ No<br>☐ Not sure
	Daily activity related to Medication-related Action Plan	4. Do you provide a record of the action plan for the patient so they can observe the progress in their treatment?<br>☐ Yes<br>☐ No<br>☐ Not sure
	Daily activities related to Intervention and Referral	5. Do you design and implement strategies to address or prevent medication-related problems?<br>☐ Yes<br>☐ No

		☐ Not sure
		6. Are you able to cooperate with other health professionals in caring for patients?<br>☐ Yes<br>☐ No<br>☐ Not sure
	Daily activities related to Documentation and Follow-up	7. To evaluate the progress of your patient's treatment, do you document the services and interventions provided?<br>☐ Yes<br>☐ No<br>☐ Not sure
		8. Do you evaluate the progress of the patient's treatment?<br>☐ Yes<br>☐ No<br>☐ Not sure
Barriers and Facilitators	Facilitators	What facilitators do you think will make the implementation of the MTM program feasible?<br>…………………………………………..
	Barriers	What barriers do you think may hinder the implementation of the MTM program in the future?<br>…………………………………………..
Additional Information	The intention to provide MTM service	1. If MTM will be implemented in the future, would you like to be an MTM provider?<br>☐ Yes<br>☐ No<br>☐ Not sure
	Availability of time to provide MTM	2. Do you think that you will have enough time to apply MTM services in the future?<br>☐ Yes<br>☐ No<br>☐ Not sure
	Availability of time and physical space for counseling	3. In your current practice, do you think that you spend enough time counseling your patients?<br>☐ Yes<br>☐ No<br>☐ Not sure
		4. Does the place where you work have a private counseling area?<br>☐ Yes<br>☐ No<br>☐ Not sure
	Accessibility of guideline in supporting the practice of Medication Therapy Review, Personal Medication Record,	5. Do you access the "Guidelines for Basic Medication in the Community Health Center" (online or hard copy) to support the review of patient treatment?<br>☐ Yes<br>☐ No<br>☐ Not sure

	and Medication-related Action Plan	6. Do you access other guidelines and drug information resources (online or hard copies)?<br>☐ Yes<br>☐ No<br>☐ Not sure
	Training needs	7. Do you think that a lack of training can hinder the implementation of MTM?<br>☐ Yes<br>☐ No<br>☐ Not sure
		8. Are you interested in learning more information about providing an MTM service?<br>☐ Yes<br>☐ No<br>☐ Not sure<br><br>9. If yes, which method do you prefer:<br>☐ online education<br>☐ live workshop
